# Supplementary material for: The Malay Literacy of Suicide Scale: A Rasch Model Validation and Its Correlation with Mental Health Literacy among Malaysian Parents, Caregivers and Teachers
Source: Healthcare (Basel). 2022 Jul 14;10(7):1304. doi: 10.3390/healthcare10071304 (PMC9317984; doi:10.3390/healthcare10071304)
Supplement: Supplementary file 1 [file healthcare-10-01304-s001.zip › S1 Table.pdf]

Table S1. Content validity of 27-item M-LOSS

Item	Expert 1	Expert 2	Expert 3	Expert 4	Expert 5	Expert 6		Experts in Agreement	I-CVI	UA
1	1	1	1	1	1	1		6	1	1
2	1	1	1	1	1	1		6	1	1
3	1	1	1	1	1	1		6	1	1
4	1	1	1	1	1	1		6	1	1
5	1	1	1	1	1	1		6	1	1
6	1	1	0	1	1	1		5	0.83	0
7	1	1	0	1	1	1		5	0.83	0
8	1	1	1	1	1	1		6	1	1
9	1	0	1	1	1	1		5	0.83	0
10	1	1	1	1	1	1		6	1	1
11	1	1	1	1	1	1		6	1	1
12	1	1	1	1	1	1		6	1	1
13	1	1	1	1	1	1		6	1	1
14	1	1	0	1	1	1		5	0.83	0
15	1	1	1	1	1	1		6	1	1
16	1	1	1	1	1	1		6	1	1
17	1	1	1	1	1	1		6	1	1
18	1	1	1	0	1	1		5	0.83	0
19	1	1	1	0	1	1		5	0.83	0
20	1	1	1	0	1	1		5	0.83	0
21	1	1	1	0	1	1		5	0.83	0
22	1	1	1	0	1	1		5	0.83	0
23	1	1	1	0	1	1		5	0.83	0
24	1	1	1	1	1	1		6	1	1
25	1	1	1	1	1	1		6	1	1
26	1	1	1	1	1	1		6	1	1
27	1	1	1	1	1	1				
								**S-CVI/Ave**	0.94	
**Proportion relevance**	1	0.96	0.89	0.78	1	1				
**Average proportion of item judged as relevance across 6 experts**							0.94	**S-CVI/UA**		0.63
