# Supplementary material for: The Malay Literacy of Suicide Scale: A Rasch Model Validation and Its Correlation with Mental Health Literacy among Malaysian Parents, Caregivers and Teachers
Source: Healthcare (Basel). 2022 Jul 14;10(7):1304. doi: 10.3390/healthcare10071304 (PMC9317984; doi:10.3390/healthcare10071304)
Supplement: Supplementary file 1 [file healthcare-10-01304-s001.zip › S2 Table.pdf]

Table S2. Face validity of 27-item M-LOSS

ITEM	R1	R2	R3	R4	R5	R6	R7	R8	R9	R10		Raters in agreement	I-FVI	UA
1	1	1	1	1	1	1	1	1	1	1		10	1	1
2	1	1	1	1	1	1	1	1	1	1		10	1	1
3	1	1	1	1	0	1	1	1	1	1		9	0.9	0
4	1	1	1	1	1	1	1	1	1	1		10	1	1
5	1	1	1	1	1	1	1	1	1	0		9	0.9	0
6	1	1	1	1	1	1	1	1	1	0		9	0.9	0
7	1	1	1	1	1	1	1	0	1	1		9	0.9	0
8	1	1	1	1	1	1	1	0	1	1		9	0.9	0
9	1	1	1	1	1	1	1	1	1	1		10	1	1
10	1	1	1	1	1	1	1	1	1	1		10	1	1
11	1	1	1	1	1	1	1	1	1	1		10	1	1
12	1	1	1	1	1	1	1	1	1	1		10	1	1
13	1	1	1	1	1	1	1	1	1	1		10	1	1
14	1	1	1	1	1	1	1	0	1	1		9	0.9	0
15	1	1	1	1	1	1	1	1	1	1		10	1	1
16	1	1	1	1	1	1	1	1	1	1		10	1	1
17	1	1	1	1	1	1	1	1	1	1		10	1	1
18	1	1	1	1	1	1	1	1	1	1		10	1	1
19	1	1	1	1	1	1	1	1	1	1		10	1	1
20	1	1	1	1	1	1	1	1	1	1		10	1	1
21	1	1	1	1	1	1	1	1	1	1		10	1	1
22	1	1	1	1	1	1	1	1	1	1		10	1	1
23	1	1	1	1	1	1	1	1	1	1		10	1	1
24	1	1	1	1	1	1	1	0	1	1		9	0.9	0
25	1	1	1	1	1	1	1	1	1	1		10	1	1
26	1	1	1	1	1	1	1	1	1	1		10	1	1
27	1	1	1	1	1	1	1	1	1	1		10	1	1
												**S-FVI/Ave**	0.97	
**Proportion clarity & comprehension**	1	1	1	1	0.96	1	1	0.85	1	0.93				
**Average proportion of items judged clarity and comprehension across the 10 raters**											0.97	**S-FVI/UA**		0.74
