# Supplementary material for: The Malay Literacy of Suicide Scale: A Rasch Model Validation and Its Correlation with Mental Health Literacy among Malaysian Parents, Caregivers and Teachers
Source: Healthcare (Basel). 2022 Jul 14;10(7):1304. doi: 10.3390/healthcare10071304 (PMC9317984; doi:10.3390/healthcare10071304)
Supplement: Supplementary file 1 [file healthcare-10-01304-s001.zip › S3 Table.pdf]

Table S3. Item fit analysis for 27-item M-LOSS

Entry no.	Total score	Count	Measure	Model S.E.	Infit		Outfit		Pt-Measure		Exact OBS%	Match EXP%	Items
					MNSQ	ZSTD	MNSQ	ZSTD	CORR.	EXP.			
18	627	750	-1.07	0.10	1.12	1.8	1.25	2.2	0.16	0.28	82.6	84.0	LOSS18
27	726	750	-3.02	0.22	0.99	0.0	0.84	-0.5	0.20	0.19	97.0	97.0	LOSS27
3	687	750	-1.90	0.14	1.02	0.3	0.90	-0.5	0.23	0.23	91.4	91.8	LOSS3
20	591	750	-0.72	0.09	1.05	1.1	1.11	1.3	0.24	0.30	79.1	79.4	LOSS20
12	675	750	-1.70	0.13	0.99	-0.1	0.97	-0.1	0.25	0.25	90.6	90.2	LOSS12
5	710	750	-2.43	0.17	0.97	-0.2	0.81	-0.9	0.26	0.21	94.9	94.8	LOSS5
19	450	750	0.32	0.08	1.10	3.4	1.13	2.8	0.26	0.36	62.9	66.5	LOSS19
1	296	750	1.30	0.08	1.11	3.5	1.17	3.4	0.27	0.38	64.5	68.4	LOSS1
13	609	750	-0.89	0.10	1.01	0.2	0.98	-0.1	0.28	0.29	81.6	81.7	LOSS13
9	490	750	0.05	0.08	1.02	0.8	1.04	0.7	0.32	0.34	69.9	69.1	LOSS9
11	634	750	-1.15	0.11	0.96	-0.6	0.94	-0.5	0.32	0.28	85.3	84.9	LOSS11
22	531	750	-0.23	0.09	1.01	0.3	1.00	0.1	0.32	0.33	72.5	72.7	LOSS22
14	374	750	0.80	0.08	1.04	1.5	1.09	2.1	0.33	0.37	62.0	65.1	LOSS14
4	614	750	-0.93	0.10	0.95	-0.8	0.92	-0.8	0.34	0.29	82.8	82.3	LOSS4
26	238	750	1.70	0.09	1.02	0.6	1.10	1.6	0.35	0.38	72.7	73.0	LOSS26
10	503	750	-0.03	0.08	0.98	-0.5	0.97	-0.4	0.35	0.34	71.9	70.0	LOSS10
17	312	750	1.19	0.08	1.03	1.0	1.03	0.7	0.35	0.38	65.3	67.3	LOSS17
8	311	750	1.20	0.08	1.03	0.9	1.02	0.5	0.36	0.38	66.5	67.3	LOSS8
2	292	750	1.32	0.08	1.02	0.7	1.01	0.1	0.36	0.38	66.9	68.7	LOSS2
23	346	750	0.97	0.08	1.00	-0.1	0.98	-0.4	0.38	0.38	65.9	65.6	LOSS23
16	462	750	0.24	0.08	0.96	-1.4	0.91	-1.8	0.40	0.35	66.9	67.2	LOSS16
6	336	750	1.04	0.08	0.96	-1.3	0.94	-1.4	0.41	0.38	67.6	66.1	LOSS6
7	313	750	1.18	0.08	0.97	-1.2	0.94	-1.2	0.41	0.38	67.2	67.2	LOSS7
15	521	750	-0.16	0.08	0.92	-2.3	0.85	-2.5	0.42	0.33	72.9	71.7	LOSS15
25	299	750	1.28	0.08	0.95	-1.8	0.92	-1.7	0.43	0.38	71.1	68.2	LOSS25
21	505	750	-0.05	0.08	0.90	-3.0	0.83	-3.0	0.44	0.34	73.2	70.2	LOSS21
24	238	750	1.70	0.09	0.91	-2.4	0.93	-1.1	0.46	0.38	76.4	73.0	LOSS24
