# Supplementary material for: The Malay Literacy of Suicide Scale: A Rasch Model Validation and Its Correlation with Mental Health Literacy among Malaysian Parents, Caregivers and Teachers
Source: Healthcare (Basel). 2022 Jul 14;10(7):1304. doi: 10.3390/healthcare10071304 (PMC9317984; doi:10.3390/healthcare10071304)
Supplement: Supplementary file 1 [file healthcare-10-01304-s001.zip › S4 Table.pdf]

Table S4. Item fit analysis for 26-item M-LOSS

Entry no.	Total score	Count	Measure	Model S.E.	Infit		Outfit		Pt-Measure		Exact OBS%	Match EXP%	Items
					MNSQ	ZSTD	MNSQ	ZSTD	CORR.	EXP.			
26	726	750	-3.07	0.22	1.00	0.1	0.89	-0.3	0.20	0.19	97.0	97.0	LOSS27
3	687	750	-1.96	0.14	1.04	0.4	0.93	-0.3	0.22	0.24	91.4	91.8	LOSS3
19	591	750	-0.76	0.09	1.07	1.3	1.14	1.5	0.24	0.31	78.2	79.5	LOSS20
18	450	750	0.28	0.08	1.11	3.8	1.16	3.2	0.26	0.36	62.2	66.8	LOSS19
12	675	750	-1.75	0.13	0.99	-0.1	0.98	-0.1	0.26	0.25	90.6	90.2	LOSS12
5	710	750	-2.49	0.17	0.97	-0.2	0.82	-0.8	0.26	0.22	94.9	94.8	LOSS5
1	296	750	1.26	0.08	1.11	3.6	1.17	3.3	0.28	0.39	63.9	68.5	LOSS1
13	609	750	-0.93	0.10	1.02	0.4	0.99	-0.1	0.29	0.30	81.7	81.8	LOSS13
9	490	750	0.01	0.08	1.04	1.1	1.06	1.0	0.32	0.35	69.9	69.4	LOSS9
11	634	750	-1.20	0.11	0.96	-0.5	0.95	-0.4	0.32	0.29	85.5	85.0	LOSS1
21	531	750	-0.28	0.09	1.02	0.5	1.01	0.2	0.32	0.34	72.3	73.0	LOSS22
14	374	750	0.76	0.08	1.04	1.7	1.09	2.2	0.33	0.38	62.4	65.4	LOSS14
4	614	750	-0.98	0.10	0.96	-0.7	0.94	-0.5	0.34	0.30	82.6	82.4	LOSS4
25	238	750	1.67	0.09	1.03	0.7	1.11	1.8	0.35	0.39	72.8	73.1	LOSS26
17	312	750	1.16	0.08	1.03	1.2	1.04	0.9	0.36	0.39	64.7	67.4	LOSS17
10	503	750	-0.08	0.08	0.98	-0.5	0.97	-0.5	0.36	0.35	72.1	70.4	LOSS10
8	311	750	1.16	0.08	1.02	0.7	1.01	0.3	0.37	0.39	66.8	67.5	LOSS8
2	292	750	1.29	0.08	1.02	0.7	1.00	0.1	0.37	0.39	67.2	68.8	LOSS2
22	346	750	0.94	0.08	1.00	-0.1	0.98	-0.3	0.39	0.38	65.8	65.9	LOSS23
16	462	750	0.20	0.08	0.96	-1.4	0.91	-1.8	0.40	0.36	67.0	67.5	LOSS16
15	521	750	-0.20	0.09	0.93	-2.1	0.86	-2.3	0.42	0.34	72.8	72.0	LOSS15
7	313	750	1.15	0.08	0.97	-1.1	0.94	-1.3	0.42	0.38	68.6	67.4	LOSS7
6	336	750	1.00	0.08	0.96	-1.4	0.94	-1.5	0.42	0.38	67.2	66.3	LOSS6
24	299	750	1.24	0.08	0.95	-1.6	0.93	-1.5	0.43	0.39	70.8	68.3	LOSS25
20	505	750	-0.09	0.08	0.90	-3.0	0.83	-3.0	0.44	0.35	74.8	70.5	LOSS21
23	238	750	1.67	0.09	0.91	-2.4	0.95	-0.8	0.46	0.39	76.6	73.1	LOSS24
