# Supplementary material for: The Malay Literacy of Suicide Scale: A Rasch Model Validation and Its Correlation with Mental Health Literacy among Malaysian Parents, Caregivers and Teachers
Source: Healthcare (Basel). 2022 Jul 14;10(7):1304. doi: 10.3390/healthcare10071304 (PMC9317984; doi:10.3390/healthcare10071304)
Supplement: Supplementary file 1 [file healthcare-10-01304-s001.zip › S5 Table.pdf]

Table S5. Principal component analysis of residual (PCAR) of the 26-item M-LOSS

		**Empirical**		**Modeled**
Total raw variance in observation	36.6	100.0%		100.0%
Raw variance explained by measures	10.6	29.0%		25.9%
Raw variance explained by persons	3.9	10.7%		9.5%
Raw variance explained by items	6.7	18.4%		16.4%
Raw unexplained variance (total)	26.0	71.0%	100.0%	74.1%
Unexplained variance in 1st contrast	2.0	5.3%	7.5%	
Unexplained variance in 2nd contrast	1.7	4.7%	6.6%	
Unexplained variance in 3rd contrast	1.5	4.2%	5.9%	
Unexplained variance in 4th contrast	1.3	4.0%	5.6%	
Unexplained variance in 5th contrast	1.3	3.5%	5.0%	
