# Supplementary material for: The Malay Literacy of Suicide Scale: A Rasch Model Validation and Its Correlation with Mental Health Literacy among Malaysian Parents, Caregivers and Teachers
Source: Healthcare (Basel). 2022 Jul 14;10(7):1304. doi: 10.3390/healthcare10071304 (PMC9317984; doi:10.3390/healthcare10071304)
Supplement: Supplementary file 1 [file healthcare-10-01304-s001.zip › S6 Table.pdf]

Table S6. Separation and reliability indexes of the 26-item M-LOSS

**Person Separation and Reliability Indexes**								
	**Score**	**Count**	**Measure**	**Error**	**Infit**		**Outfit**	
					**IMSQ**	**ZSTD**	**OMSQ**	**ZSTS**
Mean	16.0	26.0	0.76	0.51	1.00	0.0	0.99	0.0
S.D.	3.9	0.0	0.94	0.09	0.22	1.0	0.48	0.9
Real RMSE	0.52							
ADJ. SD	0.78							
Separation	1.52							
Person reliability	0.70							
**Item Separation and Reliability Indexes**								
	**Score**	**Count**	**Measure**	**Error**	**Infit**		**Outfit**	
					**IMSQ**	**ZSTD**	**OMSQ**	**ZSTS**
Mean	464.0	750.0	0.00	0.10	1.00	0.0	0.99	0.0
S.D.	154.3	0.0	1.29	0.03	0.05	1.6	0.09	1.5
Real RMSE	0.10							
ADJ. SD	1.29							
Separation	12.47							
Item reliability	0.99							
