# Supplementary material for: The Malay Literacy of Suicide Scale: A Rasch Model Validation and Its Correlation with Mental Health Literacy among Malaysian Parents, Caregivers and Teachers
Source: Healthcare (Basel). 2022 Jul 14;10(7):1304. doi: 10.3390/healthcare10071304 (PMC9317984; doi:10.3390/healthcare10071304)
Supplement: Supplementary file 1 [file healthcare-10-01304-s001.zip › S7 Table.pdf]

Table S7. Respondents' sociodemographic (n = 867)

Variable	Mean (SD)
Age (year)	43.81 (8.34)

Variable	n (%)
Role	
Parents/caretakers	560 (64.6)
Teacher	307 (35.4)
Sex	
Male	247 (28.5)
Female	620 (71.5)
Ethnicity	
Malay	755 (87.1)
Chinese	49 (5.7)
Indian	4 (0.5)
Other Bumiputera	48 (5.5)
Others	11 (1.3)
Religion	
Islam	779 (89.9)
Christian	49 (5.7)
Hindu	3 (0.3)
Buddhist	34 (3.9)
Others	2 (0.2)
Education level	
No formal education	2 (0.2)
Primary education	11 (1.3)
Secondary education	202 (23.3)
Tertiary education	652 (75.2)
Occupation sector	
Unemployed/homemaker	102 (11.8)
Government	578 (66.7)
Private	106 (12.2)
Self-employed	63 (7.3)
Pensioner	18 (2.1)
Monthly household income bracket [a]	
B40	346 (39.9)
M40	394 (45.4)
T20	127 (14.6)
School locality	
Urban	502 (58.1)
Rural	363 (41.9)
School type	
National secondary schools (SMK)	341 (39.3)

Full boarding schools (SBP)	321 (37.0)
Religious secondary schools (SMA)	205 (23.6)
Personal history of mental health	
Yes	21 (2.4)
No	846 (97.6)
Known contact with mental health issues	
Yes	160 (18.5)
No	707 (81.5)
Assisted those with mental health issues	
Yes	217 (25.0)
No	650 (75.0)
Attended formal mental health first aid training	
Yes	
No	88 (10.1)
	779 (89.9)
